# Inter-Software Reproducibility of Quantitative Values of Myocardial Blood Flow and Coronary Flow Reserve Acquired by [^13^N]NH_3_ MPI PET/CT and the Effect of Motion Correction Tools

**DOI:** 10.3390/diagnostics15050613

**Published:** 2025-03-04

**Authors:** Oscar Isaac Mendoza-Ibañez, Riemer H. J. A. Slart, Erick Alexanderson-Rosas, Tonantzin Samara Martinez-Lucio, Friso M. van der Zant, Remco J. J. Knol, Sergiy V. Lazarenko

**Affiliations:** 1 Department of Nuclear Medicine and Molecular Imaging, University Medical Center Groningen, University of Groningen, 9713GZ Groningen, The Netherlands; r.h.j.a.slart@umcg.nl (R.H.J.A.S.); t.s.martinez.lucio@umcg.nl (T.S.M.-L.); 2 Department of Biomedical Photonic Imaging, University of Twente, 7522NB Enschede, The Netherlands; 3 Department of Nuclear Cardiology, Instituto Nacional de Cardiología Ignacio Chavez, Mexico City 14080, Mexico; erickalexandersonmd@gmail.com; 4 Department of Nuclear Medicine, Northwest Clinics, 1815JD Alkmaar, The Netherlands; f.m.vander.zant@nwz.nl (F.M.v.d.Z.); r.j.j.knol@nwz.nl (R.J.J.K.); s.v.lazarenko@nwz.nl (S.V.L.)

**Keywords:** MPI, PET/CT, 13N-ammonia, reproducibility, agreement, motion-correction, DDMC

## Abstract

**Background:** The choice of software package (SP) for image processing affects the reproducibility of myocardial blood flow (MBF) values in [^13^N]NH_3_ PET/CT scans. However, the impact of motion correction (MC) tools—integrated software motion correction (ISMC) or data-driven motion correction (DDMC)—on the inter-software reproducibility of MBF has not been studied. This research aims to evaluate reproducibility among three commonly used SPs and the role of MC. **Methods:** Thirty-six PET/CT studies from patients without myocardial ischemia or infarction were processed using QPET, Corridor-4DM (4DM), and syngo.MBF (syngo). MBF and coronary flow reserve (CFR) values were obtained without motion correction (NMC) and with ISMC and DDMC. Intraclass correlation coefficients (ICC) and Bland-Altman (BA) plots were used to analyze agreement. **Results:** Good or excellent reproducibility (ICC ≥ 0.77) was found for rest-MBF values, regardless of the SPs or use of MC. In contrast, stress-MBF and CFR values presented mostly a moderate agreement when NMC was used. The RCA territory consistently had the lowest agreement in stress-MBF and CFR in the comparisons involving QPET. The use of MC, particularly DDMC, enhanced the reproducibility of most of the stress-MBF and CFR values by improving ICCs and reducing bias and limits of agreement (LoA) in BA analysis. **Conclusions:** MBF quantification agreement between SPs is strong for rest-MBF values but suboptimal for stress-MBF and CFR values. MC tools, especially DDMC, are recommended for improving reproducibility in stress-MBF assessments, although differences in SP reproducibility up to 0.77 mL/g/min in global stress-MBF and up to 0.88 in global CFR remain despite the use of MC.

## 1. Introduction

Positron emission tomography with computed tomography (PET/CT) allows for the non-invasive quantification of myocardial blood flow (MBF) and coronary flow reserve (CFR) [ratio of MBF in stress to MBF in rest] with the use of different radiotracers (i.e., ^82^Rb, [^13^N]NH_3_, [^15^O]H_2_O, and [^18^F]Flurpiridaz) [1,2]. These quantitative measurements play an important role in the evaluation of patients with suspected or confirmed ischemic heart disease (IHD) [3]. They allow for the evaluation of a broad range of pathologies, including multi-vessel disease, microvascular coronary dysfunction (MCD), microvascular angina, and myocardial infarction without obstruction of the coronary arteries [4,5,6,7]. Finally, MBF and CFR values have been shown to act as independent prognostic markers in IHD patients [8].

MBF quantification by PET/CT relies on the image reconstruction of dynamic acquisition data that follow the changes in radioactivity concentration during the acquisition (sub-sampled in a sequence of timeframes). Within these images, two types of time activity curves (TACs), namely the image-derived input function (IDIF), i.e., the radiotracer concentration in the blood pool and the tissue-TAC and the radiotracer concentration in the region of interest, are measured. Finally, using kinetic modeling, values of MBF in rest (rest-MBF) and stress (stress-MBF) are estimated, together with CFR values [9,10]. This process is performed automatically by the software package (SP) selected for image processing of dynamic PET/CT cardiac scans. Each SP presents important differences in several pivotal steps necessary for MBF quantification, such as left ventricle segmentation, myocardial wall count sampling for IDIF and tissue-TAC estimation, or myocardial-contour delineation. Moreover, it is known that dynamic image processing is prone to being impaired by the presence of motion artifacts. Several publications have demonstrated how regional and global MBF/CFR values are wrongly estimated in response to the presence of motion [either organ motion, such heart contraction, or patients’ gross body motion]. The negative effect of motion has been proven to be worse when manifested in the form of cardiac creep (i.e., non-periodic shift of the myocardial wall) [11,12].

In recent years, there has been a rapidly marked increment in the number of SPs that are commercially available for PET/CT processing, as shown by the high number of publications aimed to address the inter-software reproducibility between SPs [13,14,15,16,17]. Furthermore, the progressive interest in motion artifacts due to the proven negative effect of motion on image quality and MBF quantification has led manufacturers to incorporate different motion correction (MC) tools to overcome this issue. Both built-in software motion correction (ISMC) tools, or to develop other tools applicable within the image reconstruction process, such as a data-driven motion correction (DDMC) algorithm [18,19].

The SP selected for image processing has already been postulated to play a pivotal role in the attaining of accurate and reproducible values of MBF and CFR [15]. Consequently, the inter-software reproducibility of MBF/CFR values has already been explored in different publications. Nevertheless, differences in software reproducibility related to distinct MC approaches, such as the use of MC or not, or the type of MC tool applied, have not been considered in previous papers, and the specific role of MC tools remains unexplored. This project aims to evaluate the inter-software reproducibility of MBF/CFR values between three of the most used SPs for [^13^N]NH_3_ dynamic processing with the application of different methodologies for MC.

## 2. Materials and Methods

### 2.1. Study Population

Thirty-six patients classified as without significant myocardial perfusion defects after undergoing a cardiac PET/CT scan due to suspected IHD were retrospectively included from a clinical cohort of patients that underwent cardiac PET/CT examination in the Northwest Clinics (Alkmaar, The Netherlands) between July 2020 and February 2022. The study was approved by the institutional research board; approval of the local ethical committee was not necessary since the study did not fall within the scope of the Dutch Medical Research Involving Human Subjects Act (Section 1.b, wet medisch-wetenschappelijk onderzoek [WMO], 26 February 1998).

### 2.2. Inclusion Criteria

The following inclusion criteria were applied for the selection of patients: (1) No prior history of IHD; (2) PET/CT results in the PET/CT examination interpreted as normal by an experienced nuclear medicine physician, i.e., no significant perfusion defects by visual inspection of static images, LV-ejection fraction (LVEF) values > 55% in the gated series, and preserved global CFR ≥ 2.0 mL/min/g values in the dynamic series; (3) Diagnosis of IHD ruled out by medical consensus after clinical and imaging evaluation; (4) Follow-up from the study date to the inclusion time without relapse in symptomatology or presence of any major adverse cardiac event (MACE).

### 2.3. Image Acquisition

Images were acquired using a Biograph Vision 600 PET/CT system (Siemens Healthcare, Knoxville, TN, USA). A 25 min list-mode PET/CT scan, consisting of 12 min of rest acquisition and 12 min of stress acquisition, was performed on all patients. Pharmacologic stress was induced by either intravenous adenosine continuous infusion (0.14 mg/kg/min for 6 min) or 400 µg regadenoson (bolus in 10 s, followed by 10 mL saline). It must be noted that this is a time-efficient MPI PET/CT protocol, already validated by Opstal TSJ et al. [20], which implements a residual activity correction algorithm to eliminate the interference of [^13^N]NH^3^ residual activity in the stress phase from the rest phase. Briefly, this algorithm quantifies the residual activity from the rest injection using the first frame of the stress acquisition (acquired during 30 s before the [^13^N]NH^3^ stress injection). The IDIF and tissue-TAC obtained from the stress acquisition are then corrected by subtracting the residual activity from all frames of the decay-corrected tissue-TAC and IDIF.

### 2.4. Image Reconstruction and Processing

Static, dynamic, and 16-bin ECG-gated images were reconstructed using PSF + TOF reconstruction, a 220 × 220 matrix, zoom 2, an isotropic Gaussian 3D filter of 4 mm, 4 iterations, and 5 subsets. CT-based attenuation, scatter, decay, and random corrections were applied to all images. The quality of the registration between PET and CT was reviewed and corrected manually before the reconstruction process in case of misalignment.

Dynamic rest images were reconstructed using the first 10 min of the rest acquisition data using 25 frames (1 × 10, 12 × 5, 2 × 10, 7 × 30, 2 × 60, and 1 × 180 s), whereas dynamic stress images were reconstructed using the 10.5 min of data from stress acquisition after a delay of 90 s, using 26 frames (1 × 30, 1 × 10, 12 × 5, 2 × 10, 7 × 30, 2 × 60, and 1 × 180 s).

Image processing was performed with the use of three different commercially available SPs, namely QPET (Cedars-Sinai Medical Center, Los Angeles, CA, USA [version: 2018.0.0.232]), Corridor-4DM (Invia Medical Imaging Solutions, Ann Arbor, MI, USA [version: 2017.20]), and syngo.MBF (Siemens Healthineers, Erlangen, Germany [version: 4.3.0.101103]).

### 2.5. In-Software Motion Correction Tools

QPET ISMC: MBF quantification in QPET is based on the analysis of all time frames in the dynamic acquisition within fixed LV contour boundaries determined from the summed dynamic imaging data. QPET allows for manual ISMC for every temporal frame (i.e., frame-by-frame MC), in which the user can shift short-axis, horizontal long-axis, and vertical long-axis images in the 3D space (*X*, *Y*, and *Z* directions) [21,22]. This ISMC tool must be manually applied and independently for the rest and stress phases. For the purposes of this project, QPET ISMC was applied in the rest and stress acquisitions of all patients, both in the blood-pool phase (i.e., frames where radiotracer activity is present in the blood pool space but not yet in the myocardial tissue) and tissue phase (i.e., frames where radiotracer activity is present in the myocardial tissue and no longer in the blood-pool). In the blood-pool phase, the correction aimed to locate the radiotracer activity to the endocardial borders of the LV and uniform the spillover activity. For the tissue phase, the correction aims to align the radiotracer activity within the LV contour boundaries (LV myocardial surface).

Corridor-4DM ISMC: MBF quantification in Corridor-4DM (4DM) utilizes a myocardial image volume summed from the dynamic data, with the LV myocardial tissue activity estimated mid-wall, in the midway between the endocardial and epicardial surfaces. 4DM allows for the selection of an automatic in-software motion-correction (ISMC) tool that performs MC for every temporal frame in both the rest and stress phases. This method is an image-based algorithm that performs MC in the 3D space using normalized gradient fields, with a reported efficiency equal to manual MC [23]. This IMSC is automatically applied by the SP both for the rest and stress phases if selected.

Syngo.MBF ISMC: MBF quantification in syngo.MBF (Syngo) is based on the analysis of a summed image derived from the later frames (tissue-phase frames). The software uses a conventional cylindrical-spherical model for dynamic sampling, as described by Nekolla et al. [24]. A radial line around the mid-myocardial surface is drawn by sampling 505 radial profiles along 15 slices obtained from the model. Myocardial tissue time-activity curve data points are obtained at each timeframe as the averaged value of the mid-myocardial surface along the radial line, with a total of 505 time-activity curves being generated from the myocardium in this way. Syngo software automatically applies LV motion correction to the later frames of the acquisition by applying unique LV segmentation in every frame present in the tissue phase. An additional ISMC tool (referred to as the “high motion correction” tool) can be applied independently in both the rest and stress phases in the case of severe motion artifacts. This tool realigns the later frames using an automatic co-registration method that propagates backward from the last frame in a consecutive manner until it encounters the blood-pool phase frames [20,25,26]. In Syngo, the IMSC is automatically performed by the SP and can be independently applied in the rest or stress phases.

For the purposes of this project, automatic ISMC tools were applied both for the rest and stress phases of every scan.

### 2.6. Data-Driven Motion Correction

**Figure 1:**
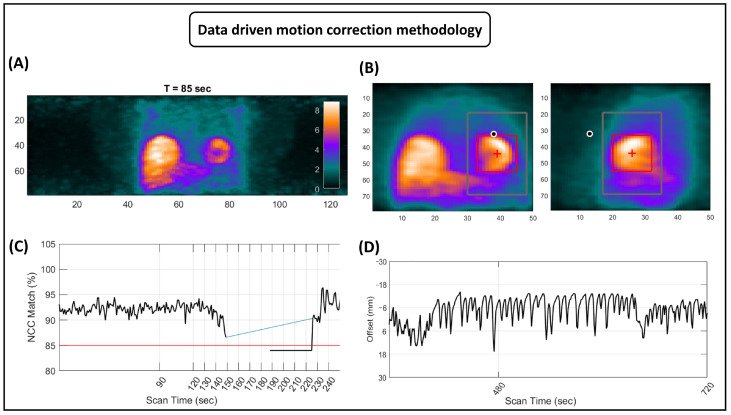
Functioning of the DDMC algorithm. (**A**) Direct Volume Histogram (DVH) constructed by the DDMC at sec = 85. (**B**) Process of heart signature (REF) detection in DDMC. The red box denoted the REF located within a predefined search range (SER) [gray box]. (**C**) DDMC normalized cross-correlation (NCC) matches in a stress acquisition up to sec = 250. The red solid line denotes the threshold of 85% established to assure reliable motion tracking. Blue solid line reflects the blood-pool period, where tracking is more difficult and sometimes unreliable. (**D**) Motion vector constructed by DDMC in the Z-direction of the second half of a stress acquisition.

A comprehensive explanation of the DDMC algorithm’s functioning is published elsewhere [27,28]. Figure 1 illustrates how the DDMC operates. As a brief description, for tracking the rigid motion of the heart (i.e., any myocardial shift caused by respiratory motion or the patient’s gross body motion), DDMC uses the PET list-mode raw data to bin 4D histo-images [3D space plus the time dimension (t)], referred to as a “Direct Volume Histogram” (DVH) (Figure 1A). A DVH is an image with enough spatial resolution (spatial localization precision of approximately three (3) cm for the positron annihilation) to locate and track the rigid translation motion of the heart. One DVH is created for each second of acquisition time, as this frequency has been proven to be the time interval that provides histo-images with a better trade-off between signal and noise (Armstrong (2022) [27]). The heart signature is a sub-image of one DVH, known as REF (Figure 1B). This REF is typically located by searching for a relatively high-intensity area within a DVH from a stable point in the scan (i.e., a period with radiotracer activity in the myocardium evenly distributed and low extra-cardiac activity). In [^13^N]NH_3_ acquisitions, a DVH near the end of dynamic acquisitions is typically used. Afterward, the REF image is compared to the other DVH images from all the acquisition time points. This comparison occurs within a predefined search range in every DVH, referred to as SER (Figure 1B). A Normalized Cross Correlation (NCC) technique is used to track the rigid heart movement (or translation) in the *X* (mediolateral), *Y* (anteroposterior), and *Z* (craniocaudal) parameter spaces. The position with the highest NCC value indicates the heart’s location at that specific time point. A threshold of 85% is used to ensure reliable motion tracking. For the untracked frames, another REF must be utilized to continue tracking. In a conventional [^13^N]NH_3_ scan, a single REF is enough to match all time points in the acquisitions, except during the blood-pool phase, right after the radiotracer’s injection, where the images change quickly. Different strategies are used to cope with these variations in the blood-pool phase (Figure 1C). In a final step, MC is implemented during sinogram creation (at its inherent resolution of 1.6 mm), producing a “motion-corrected” sinogram. In the current implementation, the DDMC focuses on axial shifts and only performs MC based on one-second resolution *Z*-axis motion (Figure 1D). This approach has been selected due to predominant cardiac motion in this direction and also to optimize computational efficiency and minimize delays in obtaining corrected images (<1 min for MC in all series of a rest/stress [^13^N]NH_3_ scan). This new motion-corrected sinogram can be ultimately reconstructed using regular protocols and processed with the use of any SP.

### 2.7. Computation of Final Variables and Statistical Analysis

**Figure 2:**
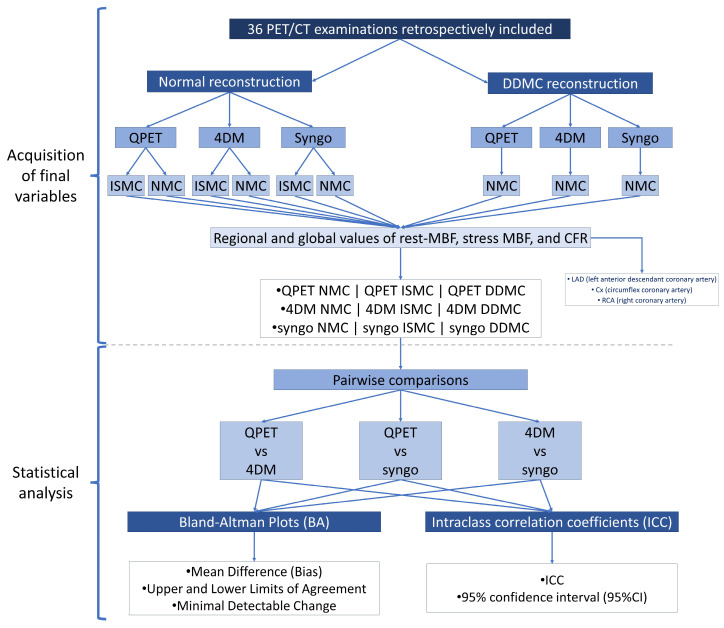
Flowchart of the methodological process for the acquisition of final variables and formal statistical analysis of the data.

Figure 2 presents an image with the general flowchart followed for the obtention of the final variables for analysis, and the specific methods that were used for statistical analysis of the data.

In more detail, the values of MBF in rest and stress per region [left-anterior descendant coronary artery (LAD), circumflex coronary artery (Cx), and right coronary artery (RCA)] and globally, as well as regional and global values of CFR, were retrieved for each SP in three different ways: (1) Using a routine reconstruction process and without the use of any ISMC tool available for each SP [i.e., no-motion correction (NMC) variables]; (2) Using a routine reconstruction process and applying the ISMC tool available to each SP (ISMC variables); (3) Performing image reconstruction with the use of the DDMC algorithm and no further ISMC in each SP (DDMC variables). The prevalence of the motion of different intensities (<3 mm, 3–6 mm, 6–9 mm, and >9 mm) in the *X*, *Y*, and *Z* directions, as referred to earlier, was estimated with the use of the motion vectors per second obtained from the DDMC algorithm.

SPs were divided into three paired groups for measuring inter-software reproducibility of rest-MBF, stress-MBF, and CFR values with the use of Bland-Altman (BA) plots and intraclass correlation coefficients (ICC). The paired comparisons were as follows: QPET versus 4DM, QPET against 4DM, and 4DM versus Syngo.

Bland-Altman (BA) plots were constructed after testing the assumptions of normality and the homoscedasticity of the data. Bias (i.e., mean difference between two methods) and limits of agreement (LoA) (i.e., range expected to include 95% of the future differences between the methods), both with their corresponding 95% confidence interval (95% CI), were calculated and plotted. Also, the minimal detectable change (MDC) (i.e., the smallest amount of change in the score detected by a method, independent of the measurement error) was calculated as one-half of the difference in the LoA range and shown in the plots.

Paired ICCs were calculated using a single measurement ICC with a two-way mixed-effect model for absolute agreement. For every ICC value, its corresponding 95% CI was calculated and reported. Agreement between SPs was categorized as “poor agreement” if ICC < 0.50, as “moderate agreement” if the ICC was between 0.51–0.75, as “good agreement” if the ICC was between 0.76–0.90, and as “excellent agreement” if ICC > 0.90.

Statistical analyses were carried out using Python 3.12.15 [25], Statsmodels (v0.14.2) [26], and the Pingouin (v0.5.5) [27] packages.

## 3. Results

### 3.1. Study Population

The characteristics of the patients are given in Table 1. The study group consisted mainly of women (72%), with a mean age of 66 years. Patients were stressed in the majority (83%) with the use of adenosine, with only six patients stressed by using regadenoson. Table 2 shows the average motion in our cohort. It can be observed how the position of the heart is accurately tracked, on average, during 473 s or more (≥78.8%) of the acquisition time of rest scans and 580 s (≥92.1%) in stress scans. These data demonstrated that high-intensity motion (≥6 mm) occurs almost exclusively in the *Z*-axis, being negligible in the other two directions (≤1% of the scanning time). Moreover, our results showed that the prevalence of motion higher than the ≈3.5 mm of the spatial resolution of the system could be placed up to ≈20% during rest scan acquisitions and up to 30% in the case of stress scans.

### 3.2. Reproducibility of NMC MBF/CFR Values

Table 3 and Table 4 show the results of the ICC and BA analysis, respectively. It can be observed how the software reproducibility varied substantially in relation to the SPs compared, the MBF/CFR values examined, and the use of MC.

When MC was not used, ICC analysis placed the reproducibility of rest-MBF values as good or excellent (ICC > 0.75), regardless of the SPs compared. Notably, 4DM and Syngo showed an excellent agreement (ICC > 0.90) in all rest-MBF values except for the LAD territory. BA analysis demonstrated that the discrepancy between all the methods was low during rest, with bias ranging from −0.16 to 0.10 mL/g/min. However, the LoA ranges were generally high for these rest-MBF paired-wise comparisons. Particularly when comparing QPET with 4DM and QPET with Syngo, the LoA ranges were always higher than 0.47 mL/g/min, reflecting an MDC >0.23 mL/g/min. In the comparison between 4DM and Syngo, although lower LoA ranges were observed, they remained >0.34 mL/g/min (MDC = 0.17 mL/g/min).

Regarding stress-MBF and CFR values, the analysis demonstrated that agreement between SPs was suboptimal when comparing NMC values, as ICC values reflected poor or moderate agreement in 9 of the 12 paired comparisons performed. Again, the comparisons of QPET and the rest of the SPs retrieved the lowest ICCs and placed the reproducibility of stress-MBF values in the Cx and RCA regions as poor (i.e., ICC < 0.50). Similarly, the RCA CFR also showed an ICC < 0.50 in the comparison of QPET versus 4DM. 4DM and Syngo obtained better ICC scores. Nevertheless, the agreement was still, in general, moderate. The findings in the BA analysis were in line with the ICC testing, as they demonstrated high discrepancies between the SPs. With an absolute bias as high as 0.79 mL/g/min observed in the RCA stress-MBF value in the comparison between QPET and Syngo. Nevertheless, the zero bias line was always inside the LoA ranges in all regional and global stress-MBF and CFR values from all SP comparisons. The LoA ranges and, consequently, the MDCs were also considerably higher than for rest-MBF values, with LoA ranges as broad as 2.99 (unitless) [RCA CFR value in the comparison QPET versus Syngo]. In the same line, BA analysis proved CFR values as with the highest LoA ranges and MDCs.

The complete BA plots for NMC MBF/CFR values are available in Supplementary Materials (Figures S1–S9).

### 3.3. Effect of the Use of MC Tools on Sofware Reproducibility

#### 3.3.1. Rest-MBF Values

The use of either ISMC or DDMC, in general, improved the ICC scores, regardless of the SPs being compared. However, and as observed in Table 3, these improvements were marginal and did not imply, most of the time, an improvement in the reported agreement. ISMC was the MC tool that improved the reported agreement the most by changing the level of agreement from good to excellent in all regional and global rest-MBF values in the comparison QPET vs. 4DM, the LAD and Cx rest-MBF values in the comparison between QPET and Syngo, and in the LAD region when comparing 4DM and Syngo. However, the use of ISMC led to a worsening of the reported agreement in the CX region of the comparison 4DM versus Syngo. On the other hand, DDMC improved the reported agreement from good to excellent only in the LAD region of the analysis between QPET and 4DM and 4DM and Syngo. Contrary to the ISMC, DDMC never caused a decrease in the reported agreement for rest-MBF values.

**Figure 3:**
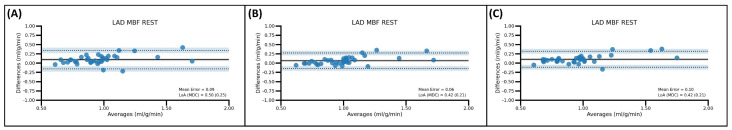
BA analysis of LAD rest-MBF values in the paired comparison of QPET and 4DM. Note how the bias (mean error) [black solid line], range of limits of agreement (LoA) [black dotted line], and minimal detectable change (MDC) are not modified in a significant extent by the use of ISMC (**B**) or DDMC (**C**) when compared to the original NMC approach (**A**).

As observed in Table 4, BA analysis showed an almost negligible effect of the MC tools on rest-MBF values, regardless of the SPs being compared. Bias proved to be similar with or without the use of ISMC or DDMC. Furthermore, although some variation was observed in the LoA ranges and MDC, this remained small (MDC variation between −0.13 and +0.03). An example of the small effect of the use of MC in rest-MBF values is shown in the BA plots in Figure 3. Complete BA plots of MBF values in rest are available in the Supplementary Materials (Figures S1–S3).

#### 3.3.2. Stress-MBF Values

During the stress phase, the use of MC tools (particularly DDMC) appeared to play an important role in improving the reproducibility between SPs, particularly when comparing QPET and the rest of the SPs. As observed in Table 3, when comparing QPET and 4DM, DDMC improved the reported agreement by ICC from poor to moderate in the Cx- and RCA-regions. Similarly, in the comparison between QPET and Syngo, the use of DDMC improved the agreement from poor to moderate in the Cx and RCA territories. In this comparison, DDMC also improved the agreement from moderate to good (ICC < 0.50 to ICC > 0.75) in the LAD region. Despite the reported agreement for global stress-MBF, values were not modified by the use of MC tools [ICC always between 0.50 and 0.75 (moderate agreement)], the ICCs’ 95% confidence intervals (95% CI) demonstrated that when using DDMC, it was assured by the obtention of ICC values with moderate or higher agreement. It has to be noted, however, that in some stress-MBF values, the use of MC tools led to a diminishment of the ICC scores. This last phenomenon was observed particularly with the use of ISMC and, more specifically, when comparing QPET and 4DM and Syngo (the LAD region) and when evaluating 4DM and Syngo (Cx and Global stress-MBF values). In this last comparison of 4DM versus Syngo, a decrement in the reported agreement took place in the RCA region, where the agreement changed from moderate to poor.

**Figure 4:**
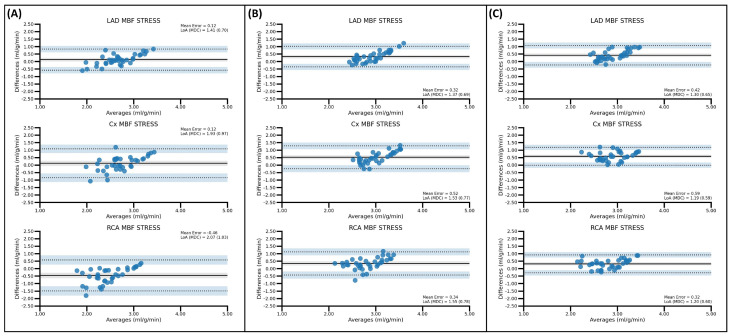
BA analysis of regional stress-MBF values in the paired comparison of QPET and 4DM. Note how the bias (mean error) [black solid line] is modified in a significant extent by the use of ISMC (**B**) or DDMC (**C**), when compared to the original NMC approach (**A**). It is important to notice how the range of the limits of agreement (LoA) [black dotted lines] and minimal detectable change (MDC) are reduced considerably when using MC tools.

As shown in Figure 4, and in line with the findings obtained in the ICC analysis, the use of MC tools, particularly DDMC, was demonstrated to improve the reproducibility of stress-MBF values by reducing the bias, the range in the LoAs, and the MDC, regardless of the SPs being compared. The only exception was the RCA stress-MBF value in the comparison between 4DM and Syngo, where the use of MC tools increased the LoA range and, consequently, the MDC substantially [increments of 0.49 mL/g/min (ISMC) and 0.31 mL/g/min (DDMC)]. MC with the use of DDMC appeared to outperform ISMC, as the ISMC approach produced smaller improvements (mean MDC reduction = 0.11 mL/g/min) than the DDMC (mean MDC reduction = 0.18 mL/g/min). Finally, even in the aforementioned region where MC increased the LoA range and MDC, DDMC increased the MDC to a lower extent, i.e., 0.16 mL/g/min, in comparison to the 0.25 mL/g/min of the ISMC approach. Complete BA plots for all regional and global MBF values for stress between the different SPs can be observed in Supplementary Materials (Figures S4–S6).

#### 3.3.3. CFR Values

The use of MC tools for the acquisition of CFR values proved to have an effect on the measured software reproducibility, although it had a smaller impact than in stress-MBF values. More specifically, the use of either ISMC or DDMC improved the ICC reported agreement from moderate to good in the Cx region and global CFR in the QPET versus 4DM comparison. In the comparison between QPET and Syngo, ISMC improved the reported agreement in the RCA region, whereas DDMC did the same in the LAD region. Finally, when comparing 4DM and Syngo, only the use of DDMC improved the interpretation of the ICC from moderate to good, although this finding should be observed carefully as the absolute increase in ICC was barely 0.02, with respect to the NMC ICC and 0.01 when compared to the ISMC ICC. Interestingly, within this comparison, the use of ISMC led to the only decrement in the reported agreement observed in the CFR values in the RCA region (good to moderate).

**Figure 5:**
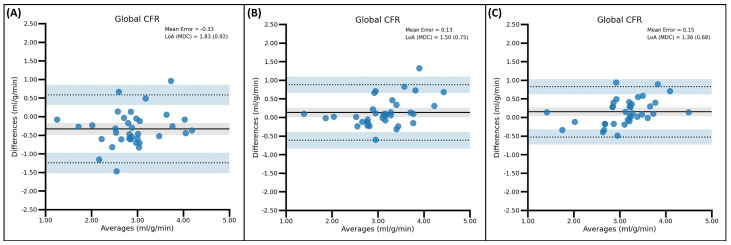
BA analysis of global CFR values in the paired comparison of QPET and 4DM. Note how the bias (mean error) [black solid line] becomes closer to the zero mean difference line after the use of ISMC (**B**) or DDMC (**C**) when compared to the original NMC approach (**A**). Note how the range in limits of agreement (LoA) [black dotted lines] and minimal detectable change (MDC) are also reduced considerably when using MC tools.

The use of ISMC or DDMC led to heterogeneous changes in the LoA ranges and MDCs of CFR values depending on the region. Reductions in the MDC of up to 0.35 [RCA-CFR (QPET vs. Syngo comparison)] after the use of ISMC and increases of up to 0.11 [RCA-CFR (QPET vs. Syngo)] after applying DDMC were observed. In any case, despite substantial reductions being achieved for some CFR values in both the LoA ranges (>0.30) and MDC (>0.15), the MDC remained ≥0.63 regardless of the SPs being compared or the MC approach. Nevertheless, it must be noted that, in general, the use of MC tools was able to bring the bias closer to the zero bias line while reducing the LoAs range and MDC, as observed in Figure 5, when compared to the NMC CFR values. The complete BA plots for CFR values between the different SPs can be observed in Supplementary Materials (Figures S7–S9).

## 4. Discussion

To our knowledge, this is the first paper that addresses the software reproducibility of quantitative values of rest-MBF, stress-MBF, and CFR, considering the newest developments of software vendors regarding the availability of diverse MC tools, both built-in software approaches (i.e., ISMC), as well as other methods aimed to achieve “during reconstruction” MC, such as the DDMC algorithm. Multiple publications have been conducted in previous years with the aim of addressing the inter-software reproducibility of MBF/CFR obtained by MPI PET/CT scans. These articles have evaluated a wide variety of SPs for MBF/CFR quantification and have included MPI PET/CT scans performed with the use of different radiotracers (i.e., ^82^Rb, [^15^O]H_2_O or [^13^N]NH_3_). Table 5 summarizes the main characteristics of these publications, including the main conclusion(s) of these articles. It must be noted that heterogeneous conclusions have been reported, most probably due to differences in radiotracer, the evaluated SPs (some papers combine SPs used in a routine clinical setting and SPs intended for research use only), and the study population [e.g., normal patients, hypertrophic cardiomyopathy, ischemia without obstruction of the coronary arteries (INOCA) patients, suspected CAD, confirmed CAD, etc.]. However, none of these papers have considered possible changes in software reproducibility introduced by the use of MC tools.

Our main results show that the decision regarding the use of MC tools and the type of MC tool to use can modify the reproducibility of [^13^N]NH_3_ PET/CT MBF quantification. This is a highly relevant finding, given the ever-increasing supply of motion correction tools and the apparent ‘discretionary’ use that physicians and/or technicians give to these methods. In the literature, only Choueiry and colleagues (2023) [35] postulated that MC in dynamic ^82^Rb PET/CT scans can improve the repeatability and reproducibility of MBF quantification. Nevertheless, in their work, they only tested the impact of MC on rest-MBF values and only used a single SP for image processing (4DM). Consequently, although they described that motion is a significant contributing to the inter- and intra-observer variability, they also depicted that the overall effect of MC was small in magnitude. Furthermore, they have faced several limitations during the study realization, including the assessment of the presence of motion, performed visually due to problems in the obtention of the frame-to-frame motion data from their MC algorithm. Moreover, the two observers agreed poorly (2 out of 280 scans) in the classification of scans with perceived motion (Cohen’s kappa score < 0.01). Nevertheless, patient motion was identified by at least one observer in 17.5% of scans (49/280). Contrary to Choueiry and colleagues, with the use of the DDMC, we have been able to estimate the prevalence of motion in our whole population and even classify it according to different intensity thresholds. Our findings suggest that significant motion (>3 mm) in the craniocaudal direction occurs in up to 20% of the acquisition time of rest acquisitions and in almost 30% of the scanning time during stress. Also, we have evaluated the effect of the use of MC not only on rest-MBF values but also on stress-MBF and CFR values. Although it must be mentioned that with respect to rest-MBF values, our analysis demonstrated similar findings as Choueiry et al., as the use of MC tools led to a discrete improvement in ICC scores and BA parameters (bias, LoA, MDC).

Perhaps the most important finding in our study is the key role that the use of MC tools appears to have in improving the reproducibility of stress-MBF and CFR values. As stated earlier in the text, MC tools have the capacity of increasing not only the ICC values but also upgrading the level of agreement derived from these values. This was achieved in 20.8% of the stress-MBF and CFR variables for ISMC and 37.5% in the case of DDMC. Interestingly, the use of MC tools, particularly ISMC, also showed the capacity to decrease the level of agreement of stress-MBF and CFR values, something that happened in approximately 8% of the cases. However, there is an explanation for this behavior. Each ISMC tool is software-specific and differs in the way they detect and correct for motion. Moreover, each SP has their unique process for MBF quantification, including different techniques for LV segmentation, myocardial wall detection, or myocardial contours delineation. All these differences ultimately make every SP prone to the effect of motion to a different extent. It is expected that the effect of the motion correction would be different for each SP. As can be observed in the Online Supplementary Materials (Figure S10), if the effect size of the MC tool differs considerably in each SP, the differences in the MBF/CFR values higher than the original differences measured in the NMC MBF/CFR values can be obtained. MC tools also proved to be highly relevant for diminishing the wideness of the LoA ranges and, therefore, for the decreasing of the MDC in all stress-MBF and CFR values. The relevance of the LoA range and the MDC parameters has been extensively described elsewhere [36], but basically, even with a zero bias, a substantially wide range in the LoA, with a consequently high MDC, can imply that the methods cannot be used interchangeably due to extreme systematic differences between them. In our results, for instance, a LoA range of 2.99, with an MDC of 1.5, was observed in the RCA CFR value of the comparison QPET versus Syngo. This would mean that if a patient undergoes an MPI PET/CT scan today with a reported CFR of 2.5 in the RCA region by analysis with QPET. And the same patient returns months after that to the hospital for a new MPI PET/CT scan due to symptom persistence, but this time analysed using Syngo software. Even a CFR value of 1 in the RCA region would not allow us to conclude the scan as abnormal, as we will not be sure if the difference is a consequence of measurement error or implies a real change. The use of ISMC can diminish on average 0.18 units the MDC in the comparison QPET vs. 4DM (up to 0.33 for the Cx CFR), but also in the comparison between QPET and Syngo (up to 0.35 for the RCA CFR). On the other hand, the use of DDMC appears to be able to diminish, on average, 0.20 units for the MDC in the comparison QPET vs. 4DM (up to 0.43 mL/g/min for RCA stress-MBF values) and to decrease an average of 0.17 units for the MDC in the comparison between QPET and Syngo (up to 0.31 mL/g/min for LAD stress-MBF values).

Despite the abovementioned information, our findings suggest that considerably high systematic differences are still present between the different SPs in stress-MBF and CFR values, regardless of the use of some of the most state-of-the-art motion correction tools. This must be carefully considered when interpreting quantitative MBF/CFR values. Mostly, in the context of multi-centric projects or in the case of serial evaluation of patients, scenarios where image processing with the use of different SPs is often needed/used.

Regarding the software reproducibility in NMC MBF/CFR values, two previous publications have evaluated the software reproducibility of PET/CT quantitative values comparing the same SPs (i.e., QPET, 4DM, and Syngo). Unfortunately, in both cases, the studies were conducted using PET/CT examinations performed using ^82^Rb as the radiotracer. The fact that we have used a different radiotracer ([^13^N]NH_3_) could potentially explain by itself any difference observed in these publications. Moreover, both Oliveira and colleagues [31] and Byrne et al. [33] analyzed reproducibility by testing differences in mean MBF/CFR values between the methods. Compared to their approach, we tested reproducibility by calculating ICC values using a single measurement ICC with a two-way mixed-effect model for measuring absolute agreement as described by Koo and Li (2016) [37]. In this way, measuring precisely the level of agreement in which two different methods (i.e., SPs) assign the same score (i.e., the same MBF/CFR value) to the same patient. However, similarly to us, Byrne and colleagues performed BA analysis. Although the biases they reported are substantially different from ours, the reported LoAs behaved similarly to those in our study. With small LoA ranges during rest but high LoA ranges in stress-MBF and CFR values (i.e., LoA range >1.12).

The inter-software reproducibility of MBF/CFR values obtained with [^13^N]NH_3_ has been studied in four previous papers with discrepant results [29,30,32,34]. However, important differences regarding the studies set up should be noted. For instance, Nesterov et. al. (2021) [34] only examined the reproducibility of stress-MBF values and included only patients with hypertrophic cardiomyopathy. These factors can explain the particularity of the results observed in their study. Slomka et al. (2012) [29] reported excellent correlation in global MBF and CFR values obtained by QPET, Syngo, and PMOD software. And concluded that normality thresholds were interchangeable between SPs, as the mean global MBF and CFR values were not significantly different between them. Similarly, Yalcin et al. (2018) [30] reported excellent agreement in global rest-MBF, global stress-MBF, and global CFR values obtained by QPET and PMOD software for suspected-CAD patients. Nevertheless, it should be noted that both papers present the major study limitation of concluding inter-software agreement via linear regression and based on correlation values. Multiple papers in the literature suggested using different tools, such as BA-plots or ICC values, that better reflect the agreement between multiple evaluators. Furthermore, in the study from Slomka and colleagues, MBF agreement was evaluated conjunctly for rest- and stress-MBF values. Our results demonstrate that the reproducibility of MBF values differs considerably if acquired at rest or in stress. Finally, Slomka and colleagues excluded patients with high spillover fractions (>0.65) in any coronary territory from the final analysis. As stated in their “Discussion” section, the main cause of this high spillover is the presence of significant patient motion. The exclusion of these patients may have caused the overestimation of the agreement between SPs when compared to our findings, as patients with significant motion artifacts were excluded according to their inclusion criteria. In contrast, in our study group, ten patients were included with spillover fractions >0.65 in at least one coronary region. And, as observed in Table 2, there was a substantial presence of motion within the acquisition time of our dynamic PET/CT rest and stress scans, particularly in the *Z*-axis (craniocaudal direction) where the respiratory motion most probably caused the rigid displacement of the myocardial wall. Complete regional and global spillover fractions for all our cohorts are provided in the Online Supplementary Materials (Table S1). Lastly, in 2018, Monroy-Gonzalez and colleagues [32] explored the agreement in MBF and CFR quantification between QPET, Syngo, and Carimas SPs. They evaluated the reproducibility in three groups: normal perfusion, patients with myocardial ischemia, and patients with myocardial infarction. Custom biplots were constructed to categorize adequate and non-adequate agreement based on the difference of MBF/CFR values and the ICC value. A difference in MBF/CFR values up to 20% combined with an ICC ≥0.75 was considered as adequate agreement. They concluded that, in patients with normal perfusion, measurements of global stress-MBF values were comparable only for the pair of Carimas and Syngo, but not for the other SPs. They also found that global rest-MBF and CFR values presented an adequate agreement regardless of the SPs being compared. At the regional level, there was marked heterogeneity in the reported levels of agreement. In summary, inadequate agreement was found for all vessels in two of the three pairwise comparisons of rest- and stress-MBF, whereas for CFR values, the RCA territory was consistently inadequate. Similarly to Monroy-Gonzalez and colleagues, our results showed good agreement for rest-MBF values in all SPs. Also, the RCA territory was the territory with the lowest agreement in two of the three paired comparisons of CFR values. However, contrary to them, we did not find adequate agreement between SPs in CFR values. Finally, along the same line as Monroy-Gonzalez et al., our results demonstrated that stress-MBF values presented the lowest agreement among all SPs.

### Limitations

There are some limitations in this study. First, it is a retrospective study with a relatively low sample size. Secondly, this paper only addressed the inter-software reproducibility in patients with suspected IHD but without the presence of significant perfusion abnormalities. It is possible, in accordance with that previously described in the literature, that the agreement in MBF/CFR values will be different in patients with myocardial ischemia, myocardial infarction, or even patients with normal perfusion but with the presence of MACEs at follow-up (suspected CMD patients). Finally, our cohort included both patients pharmacologically stressed with adenosine and regadenoson; however, differences in the reproducibility of MBF/CFR values depending on the stressor agent were not tested due to the low number of patients stressed with regadenoson in our cohort (6).

## 5. Conclusions

In patients with normal MBF and CFR, the inter-software agreement in MBF quantification among SPs is different between rest-MBF, stress-MBF, and CFR values. MC tools, particularly DDMC, have the potential to improve the agreement between SPs in stress MBF values, where the agreement is the lowest. Careful interpretation must be carried out when comparing MBF/CFR values acquired by different SPs, as changes up to 0.77 mL/g/min in global MBF values in stress and up to 0.88 mL/g/min in global CFR values can be attributed to systematic differences between the methods, despite the use of MC.

## Figures and Tables

**Table 1:** Patient characteristics.

Characteristic	*N* = 36
Sex—*n* (%)	10/36 male (28%)
Age—mean (SD)	66 years (±12)
Stress-agent—*n* (%)	Adenosine: 30 (83%) Regadenoson: 6 (17%)
Global MBF in rest—mean (±SD)	1.02 mL/g/min (±0.25)
Global MBF in stress—mean (±SD)	2.65 mL/g/min (±0.46)
Global CFR—mean (±SD)	2.72 mL/g/min (±0.62)

**Table 2:** Characteristics of heart rigid motion for the whole patient cohort according to the DDMC’s motion vectors.

Phase	Axis	Seconds with Reliable Motion Tracking Sec (%)	Motion < 3 mm Sec (%)	Motion 3–6 mm Sec (%)	Motion 6–9 mm Sec (%)	Motion > 9 mm Sec (%)
Rest	*X*	518 (86.3)	472 (91.3)	46 (8.5)	1 (0.2)	0 (0.0)
	*Y*	473 (78.8)	453 (95.9)	19 (4.0)	0 (0.1)	0 (0.0)
	*Z*	582 (97.0)	468 (80.4)	64 (10.9)	28 (4.9)	22 (3.8)
Stress	*X*	602 (95.6)	578 (96.1)	24 (3.9)	0 (0.0)	0 (0.0)
	*Y*	580 (92.1)	549 (94.7)	30 (5.2)	1 (0.2)	0 (0.0)
	*Z*	624 (99.0)	453 (72.6)	84 (13.5)	50 (8.0)	37 (5.9)

**Table 3:** Intraclass correlation coefficient (ICC) values for all paired-wise comparisons.

	QPET vs. 4DM ICC (95% CI)			QPET vs. Syngo ICC (95% CI)			4DM vs. Syngo ICC (95% CI)		
	NMC	ISMC	DDMC	NMC	ISMC	DDMC	NMC	ISMC	DDMC
LAD MBF Rest	0.87 (0.76–0.93)	0.91 ** **(0.83–0.95)**	0.91 ** **(0.84–0.96)**	0.89 (0.79–0.94)	**0.90** **(0.82–0.95)**	0.89 (0.79–0.94)	0.90 (0.82–0.95)	0.93 ** **(0.87–0.96)**	0.92 ** (0.84–0.96)
Cx MBF Rest	0.87 (0.76–0.93)	0.93 ** **(0.87–0.97)**	0.84 (0.72–0.92)	0.88 (0.77–0.94)	0.91 ** **(0.84–0.96)**	0.85 (0.73–0.92)	0.93 (0.87–0.97)	**0.94** **(0.88–0.97)**	0.93 (0.87–0.97)
RCA MBF Rest	0.77 (0.59–0.87)	0.91 ** **(0.83–0.95)**	0.82 (0.67–0.90)	0.81 (0.67–0.90)	**0.85** **(0.73–0.92)**	0.82 (0.68–0.90)	0.91 (0.83–0.95)	0.84 **^˅˅^** (0.71–0.92)	**0.95** **(0.91–0.97)**
Global MBF Rest	0.88 (0.78–0.94)	0.93 ** **(0.86–0.96)**	0.90 (0.82–0.95)	0.89 (0.79–0.94)	0.92 ** **(0.84–0.96)**	0.89 (0.80–0.94)	0.92 (0.84–0.96)	0.93 (0.86–0.96)	**0.94** **(0.88–0.97)**
LAD MBF Stress	**0.62** **(0.37–0.79)**	0.53 (0.24–0.73)	0.53 (0.25–0.73)	0.56 (0.28–0.75)	0.59 (0.33–0.77)	0.78 ** **(0.61–0.88)**	0.57 (0.30–0.76)	0.59 (0.33–0.77)	**0.61** **(0.36–0.78)**
Cx MBF Stress	0.38 (0.06–0.62)	0.45 (0.14–0.67)	0.61 ** **(0.36–0.78)**	0.46 (0.16–0.68)	0.61 ** (0.35–0.78)	0.63 ** **(0.39–0.79)**	0.67 (0.44–0.81)	0.63 (0.38–0.79)	**0.72** **(0.51–0.85)**
RCA MBF Stress	0.34 (0.02–0.60)	0.48 (0.18–0.70)	0.68 ** **(0.46–0.82)**	0.42 (0.11–0.65)	0.59 ** **(0.33–0.77)**	0.57 ** (0.31–0.76)	**0.72** **(0.52–0.85)**	0.49 **^˅˅^** (0.20–0.70)	0.71 (0.50–0.84)
Global MBF Stress	0.51 (0.22–0.71)	0.57 (0.30–0.76)	**0.72** **(0.51–0.85)**	0.51 (0.22–0.72)	0.61 (0.36–0.78)	**0.73** **(0.52–0.85)**	0.69 (0.48–0.83)	0.65 (0.42–0.81)	**0.73** **(0.53–0.85)**
LAD CFR	**0.79** **(0.63–0.89)**	**0.79** **(0.63–0.89)**	0.78 (0.62–0.88)	0.71 (0.50–0.84)	0.70 (0.49–0.84)	0.76 ** **(0.57–0.87)**	0.71 (0.51–0.84)	**0.73** **(0.54–0.85)**	0.69 (0.47–0.83)
Cx CFR	0.71 (0.50–0.84)	0.87 ** **(0.76–0.93)**	0.76 ** (0.57–0.87)	0.64 (0.40–0.80)	**0.74** **(0.55–0.86)**	0.73 (0.54–0.86)	0.77 (0.59–0.87)	0.75 (0.56–0.86)	**0.78** **(0.61–0.88)**
RCA CFR	0.57 (0.31–0.76)	**0.73** **(0.53–0.85)**	0.58 (0.31–0.76)	0.45 (0.15–0.68)	0.68 ** **(0.46–0.82)**	0.41 (0.10–0.65)	**0.78** **(0.61–0.88)**	0.70 **^˅˅^** (0.49–0.84)	**0.78** **(0.62–0.88)**
Global CFR	0.75 (0.57–0.87)	0.82 ** (0.68–0.91)	0.85 ** **(0.72–0.92)**	0.66 (0.43–0.81)	**0.74** **(0.55–0.86)**	**0.74** **(0.54–0.86)**	0.74 (0.55–0.86)	0.75 (0.56–0.86)	0.76 ** **(0.58–0.87)**

In bold are highlighted the highest ICC per region per paired comparison. ** The interpretation of ICC improved after the use of the motion correction method. ^˅˅^ The interpretation of ICC worsened after the use of the motion correction method.

**Table 4:** Differences in bias, LoA, and MDC values from Bland-Altman analysis introduced by the use of ISMC and DDMC when compared to original NMC values.

	QPET vs. 4DM									QPET vs. Syngo									4DM vs. Syngo									
	NMC			ISMC			DDMC			NMC			ISMC			DDMC			NMC			ISMC				DDMC		
	Bias	LoA Range	MDC	Bias	LoA Range	MDC	Bias	LoA Range	MDC	Bias	LoA Range	MDC	Bias	LoA Range	MDC	Bias	LoA Range	MDC	Bias	LoA Range	MDC	Bias	LoA Range	MDC	Bias		LoA Range	MDC
LAD MBF Rest (mL/g/min)	0.09	0.50	0.25	0.06	0.42	0.21	0.10	0.42	0.21	−0.06	0.47	0.23	−0.04	0.45	0.23	−0.03	0.49	0.24	−0.16	0.39	0.20	−0.11	0.33	0.17	−0.13		0.37	0.18
Cx MBF Rest (mL/g/min)	0.10	**0.51**	0.26	0.10	0.37	0.18	0.11	**0.59**	0.29	−0.01	0.49	0.24	0.03	0.43	0.21	0.03	**0.56**	0.28	−0.11	0.34	0.17	−0.08	0.34	0.17	−0.09		0.34	0.17
RCA MBF Rest (mL/g/min)	0.04	**0.68**	0.34	0.08	0.42	0.21	0.09	**0.66**	0.33	−0.04	**0.63**	0.32	−0.04	**0.59**	0.29	0.05	**0.67**	0.34	−0.08	0.40	0.20	−0.12	**0.54**	0.27	−0.04		0.29	0.14
Global MBF Rest (mL/g/min)	0.10	0.48	0.24	0.09	0.38	0.19	0.11	0.45	0.23	−0.04	0.47	0.23	−0.02	0.42	0.21	0.01	0.48	0.24	−0.13	0.37	0.18	−0.11	0.35	0.17	−0.11		0.32	0.16
LAD MBF Stress (mL/g/min)	0.12	**1.41**	0.70	**0.32**	**1.37**	0.69	**0.42**	**1.30**	0.65	−**0.37**	**1.78**	0.89	0.03	**1.55**	0.78	0.04	**1.16** **	0.58	**−0.49**	**1.35**	0.68	**−0.30**	**1.15**	0.57	**−0.38**		**1.24**	0.62
Cx MBF Stress (mL/g/min)	0.12	**1.93**	0.96	**0.52**	**1.53** **	0.77	**0.59**	**1.19** **	0.59	−**0.28**	**2.09**	1.04	**0.25**	**1.61** **	0.81	0.14	**1.49** **	0.74	**−0.40**	**1.26**	0.63	**−0.27**	**1.20**	0.60	**−0.44**		**1.25**	0.62
RCA MBF Stress (mL/g/min)	−**0.46**	**2.07**	1.03	**0.34**	**1.55** **	0.78	**0.32**	**1.20** **	0.60	**−0.79**	**2.30**	1.15	−0.08	**1.78** **	0.89	−0.04	**1.99**	0.99	**−0.33**	**1.23**	0.61	**−0.42**	**1.72** ^⌂⌂^	0.86	**−0.36**		**1.54**	0.77
Global MBF Stress (mL/g/min)	−0.04	**1.68**	0.84	**0.36**	**1.33**	0.67	**0.43**	**1.02** **	0.51	**−0.42**	**1.91**	0.95	0.07	**1.53**	0.77	0.06	**1.30** **	0.65	**−0.38**	**1.19**	0.59	**−0.28**	**1.15**	0.57	**−0.37**		**1.19**	0.59
LAD CFR (mL/g/min)	−0.13	**1.62**	0.81	0.19	**1.57**	0.79	0.17	**1.68**	0.84	−0.17	**1.86**	0.93	0.20	**1.86**	0.93	0.20	**1.69**	0.84	−0.04	**1.63**	0.81	0.00	**1.47**	0.73	0.03		**1.71**	0.85
Cx CFR (mL/g/min)	−0.18	**1.93**	0.96	**0.22**	**1.26** **	0.63	**0.30**	**1.59**	0.80	−**0.25**	**2.05**	1.02	0.20	**1.72**	0.86	0.11	**1.71**	0.85	−0.07	**1.47**	0.73	−0.02	**1.53**	0.77	−0.18		**1.37**	0.69
RCA CFR (mL/g/min)	−**0.67**	**2.72**	1.36	0.16	**2.10** **	1.05	0.15	**2.57**	1.28	**−0.75**	**2.99**	1.50	0.10	**2.29** **	1.15	−0.09	**3.22**	1.61	−0.08	**1.83**	0.92	−0.06	**1.93**	0.96	−0.25		**1.63**	0.81
Global CFR (mL/g/min)	−**0.33**	**1.83**	0.92	0.13	**1.50**	0.75	0.15	**1.36** **	0.68	**−0.32**	**2.01**	1.00	0.17	**1.76**	0.88	0.10	**1.76**	0.88	0.02	**1.63**	0.81	0.04	**1.41**	0.70	−0.05		**1.47**	0.73

In bold are highlighted the regions where there was a bias ≥ 0.2 units between the compared SPs. Bold numbers that are double underlined point to the region where the Limits of Agreement (LoA) range was higher than 0.5 units, and therefore the minimal detectable change (MDC) was higher than 0.25 units. ** Regions where MC improved the LoA range by >0.40 units. ^⌂⌂^ Regions where MC worsened the LoA range by >0.40 units.

**Table 5:** Summary of the main characteristics of the articles that have evaluated the inter-software reproducibility of MBF and/or CFR values from PET/CT MPI scans.

Authors	Journal	Year	Tracer	Software Packages	Population	Conclusions
Slomka et al. [29]	*J. Nucl. Med.*	2012	[^13^N]NH_3_	QPET vs. SyngoMBF vs. PMOD	Patients with low likelihood for CAD and patients with myocardial ischemia (visual)	Different implementations of 1TCM and 2TCM for dynamic [^13^N]NH_3_ PET demonstrate excellent correlation in MBF and MFR for each vascular territory. Reference limits appear to be interchangeable between different methods of analysis.
Dekemp et al. [13]	*J. Nucl. Med.*	2013	^82^Rb	QPET vs. SyngoMBF vs. FlowQuant	Patients with known or suspected CAD	The comparison of the three SPs (syngoMBF, FlowQuant, and QPET) shows good agreement between MBF and MFR values, with consistent identification of abnormal stress flow and flow reserve in most patients.
Tahari et al. [16]	*Eur. J. Nucl. Med. Mol. Imaging*	2014	^82^Rb	4DM (FA and kinetic modeling) vs. 4DM (ROI) vs. MunichHeart (MH)	Patients with known or suspected CAD	Quantitative assessment of rest and stress MBF with ^82^Rb PET is dependent on the software and methods used, whereas CFR appears to be more comparable. Follow-up and treatment assessment should be done with the same software and method.
Dunet et al. [14]	*J. Nucl. Cardiol.*	2015	^82^Rb	PMOD vs. FlowQuant	Patients with known or suspected CAD	Concordance between SPs was excellent for MBF and MFR. Both SPs can be used interchangeably for quantification in ^82^Rb cardiac PET.
Sunderland et al. [15]	*J. Nucl. Cardiol.*	2015	^82^Rb	SyngoMBF vs. FlowQuant vs. PMOD	Patients with suspected CAD (low likelihood)	Even when using the same TCM with identical modeling assumptions, different software generates statistically different MBF and MFR values.
Yalcin et al. [30]	*J. Nucl. Cardiol.*	2019	[^13^N]NH_3_	QPET vs. PMOD	Hypertrophic cardiomyopathy	SPs cannot be used interchangeably for MBF analyses in HCM patients.
Oliveira, J.B., Sen, Y.M. and Wechalekar, K. [31]	*J. Nucl. Cardiol.*	2018	^82^Rb	QPET vs. 4DM vs. SyngoMBF	Patients referred for an ^82^Rb PET/CT scan	Users should be cautious when using different SPs, as systematic differences amongst them may introduce wider quantitative variation of clinical significance.
Monroy-Gonzalez et al. [32]	*J. Nucl. Cardiol.*	2020	[^13^N]NH_3_	QPET vs. SyngoMBF vs. Carimas	3 groups based on SS: Normal = SSS <4 Ischemia = SDS > 2 and SRS < 4 Infarction = SRS ≥ 4	Worst agreement in global stress MBF and MFR and in patients with ischemia. Discrepancies were shown to be regionally dependent. Reproducibility between SPs should not be assumed.
Byrne et al. [33]	*J. Nucl. Cardiol.*	2021	^82^Rb	QPET vs. 4DM vs. SyngoMBF	Healthy volunteers	The reproducibility of MFR varied for the different software. MBF was analyzed with syngo. MBF and QGS may be mutually comparable, but 4DM may be preferred for analyses due to possibly higher scan-to-scan repeatability.
Nesterov et al. [34]	*J. Nucl. Cardiol.*	2022	[^13^N]NH_3_	Carimas vs. FlowQuant vs. PMOD	Hypertrophic cardiomyopathy	Carimas can be used interchangeably with both PMOD and FlowQuant for 1TCM implementation on all levels (global, regional, and segmental).
Chuxin et al. [17]	*Res. Sq.* *(Pre-print)*	2023	[^13^N]NH_3_	PMOD vs. HeartSee	INOCA (suspected CAD but no obstruction by ICA)	SPs could not be used interchangeably for absolute quantification but can be used in non-obstructive CAD patients for clinical diagnosis.

PET/CT = positron emission tomography with computed tomography, MBF = myocardial blood flow, MFR/CFR = myocardial/coronary flow reserve, SP = software package, FA = factor analysis, ROI = region of interest, INOCA = ischemia without obstructed coronary arteries, ICA = invasive coronary angiography, CAD = coronary artery disease, TCM = tissue compartmental model.

## Data Availability

Data is unavailable due to privacy or restrictions.

## Supplementary Materials

The following supporting information can be downloaded at: https://www.mdpi.com/article/10.3390/diagnostics15050613/s1, Figure S1: QPET vs. 4DM: BA analysis of rest-MBF values; Figure S2: QPET vs. Syngo: BA analysis of rest-MBF values; Figure S3: 4DM vs. Syngo: BA analysis of rest-MBF values; Figure S4: QPET vs. 4DM: BA analysis of stress-MBF values; Figure S5: QPET vs. Syngo: BA analysis of stress-MBF values; Figure S6: 4DM vs. Syngo: BA analysis of stress-MBF values; Figure S7: QPET vs. 4DM: BA analysis of CFR values; Figure S8: QPET vs. Syngo: BA analysis of CFR values; Figure S9: 4DM vs. Syngo: BA analysis of CFR values; Figure S10: Decrease in ICC after the use of MC; Table S1: Spill-over Fractions.

## References

[1] Klein R., Beanlands R.S.B., Dekemp R.A. (2010). Quantification of myocardial blood flow and flow reserve: Technical aspects. J. Nucl. Cardiol..

[2] Yoshinaga K., Manabe O., Tamaki N. (2018). Absolute quantification of myocardial blood flow. J. Nucl. Cardiol..

[3] Ngo V., Martineau P., Harel F., Pelletier-Galarneau M. (2022). Improving Detection of CAD and Prognosis with PET/CT Quantitative Absolute Myocardial Blood Flow Measurements. Curr. Cardiol. Rep..

[4] Al-Mallah M.H., Nayfeh M., Alrifai M. (2024). The role of cardiac PET in diagnosis and prognosis of patients with ischemia with no obstructive coronary arteries (INOCA). Am. Heart J. Plus Cardiol. Res. Pract..

[5] Pelletier-Galarneau M., Dilsizian V. (2020). Microvascular Angina Diagnosed by Absolute PET Myocardial Blood Flow Quantification. Curr. Cardiol. Rep..

[6] Schindler T.H., Dilsizian V. (2020). Coronary Microvascular Dysfunction. JACC Cardiovasc. Imaging.

[7] Ziadi M.C., Dekemp R.A., Williams K., Guo A., Renaud J.M., Chow B.J., Klein R., Ruddy T.D., Aung M., Garrard L. (2012). Does quantification of myocardial flow reserve using rubidium-82 positron emission tomography facilitate detection of multivessel coronary artery disease?. J. Nucl. Cardiol..

[8] Alahdab F., Al Rifai M., Ahmed A.I., Al-Mallah M.H. (2023). Advances in Digital PET Technology and Its Potential Impact on Myocardial Perfusion and Blood Flow Quantification. Curr. Cardiol. Rep..

[9] Driessen R.S., Raijmakers P.G., Stuijfzand W.J., Knaapen P. (2013). Myocardial perfusion imaging with PET. Imaging Med..

[10] Sohn J.H., Behr S.C., Pampaloni M.H., Seo Y. (2023). Quantitative Assessment of Myocardial Ischemia With Positron Emission Tomography. J. Thorac. Imaging.

[11] Hunter C.R.R.N., Klein R., Beanlands R.S., Dekemp R.A. (2016). Patient motion effects on the quantification of regional myocardial blood flow with dynamic PET imaging. Med. Phys..

[12] Koenders S.S., Jager P.L., Ottervanger J.P., Slump C.H., van Dalen J.A. (2019). Impact of regadenoson-induced myocardial creep on dynamic Rubidium-82 PET myocardial blood flow quantification. J. Nucl. Cardiol..

[13] de Kemp R.A., Declerck J., Klein R., Pan X., Nakazato R., Tonge C., Arumugam P., Berman D.S., Germano G., Beanlands R.S. (2013). Multisoftware Reproducibility Study of Stress and Rest Myocardial Blood Flow Assessed with 3D Dynamic PET/CT and a 1-Tissue-Compartment Model of ^82^Rb Kinetics. J. Nucl. Med..

[14] Dunet V., Klein R., Allenbach G., Renaud J., Dekemp R.A., Prior J.O. (2016). Myocardial blood flow quantification by Rb-82 cardiac PET/CT: A detailed reproducibility study between two semi-automatic analysis programs. J. Nucl. Cardiol..

[15] Sunderland J.J., Pan X.-B., Declerck J., Menda Y. (2015). Dependency of cardiac rubidium-82 imaging quantitative measures on age, gender, vascular territory, and software in a cardiovascular normal population. J. Nucl. Cardiol..

[16] Tahari A.K., Lee A., Rajaram M., Fukushima K., A Lodge M., Lee B.C., Ficaro E.P., Nekolla S., Klein R., Dekemp R.A. (2014). Absolute myocardial flow quantification with 82Rb PET/CT: Comparison of different software packages and methods. Eur. J. Nucl. Med..

[17] Zhang C., Wang R., Hu Y., Huangfu S., Yao Q., Li S. (2023). A cross-comparison between PMOD and HeartSee for absolute quantification of myocardial blood flow in PET imaging. Res. Square.

[18] Han Y., Ahmed A.I., Hayden C., Jung A.K., Saad J.M., Spottiswoode B., Nabi F., Al-Mallah M.H. (2022). Change in positron emission tomography perfusion imaging quality with a data-driven motion correction algorithm. J. Nucl. Cardiol..

[19] Lu Y., Liu C. (2018). Patient motion correction for dynamic cardiac PET: Current status and challenges. J. Nucl. Cardiol..

[20] Opstal T.S.J., Knoll R.J.J., Cornel J.H., Wondergem M., van der Zant F.M. (2018). Myocardial blood flow and myocardial flow reserve values in 13N–ammonia myocardial perfusion PET/CT using a time-efficient protocol in patients without coronary artery disease. Eur. J. Hybrid Imaging.

[21] Kuronuma K., Wei C.C., Singh A., Lemley M., Hayes S.W., Otaki Y., Hyun M.C., Van Kriekinge S.D., Kavanagh P., Huang C. (2024). Automated Motion Correction for Myocardial Blood Flow Measurements and Diagnostic Performance of 82Rb PET Myocardial Perfusion Imaging. J. Nucl. Med..

[22] Nakazato R., Berman D.S., Dey D., Le Meunier L., Hayes S.W., Fermin J.S., Cheng V.Y., Thomson L.E.J., Friedman J.D., Germano G. (2012). Automated quantitative Rb-82 3D PET/CT myocardial perfusion imaging: Normal limits and correlation with invasive coronary angiography. J. Nucl. Cardiol..

[23] Lee B.C., Moody J.B., Poitrasson-Rivière A., Melvin A.C., Weinberg R.L., Corbett J.R., Murthy V.L., Ficaro E.P. (2020). Automated dynamic motion correction using normalized gradient fields for 82rubidium PET myocardial blood flow quantification. J. Nucl. Cardiol..

[24] Nekolla S.G., Miethaner C., Nguyen N., Ziegler S.I., Schwaiger M. (1998). Reproducibility of polar map generation and assessment of defect severity and extent assessment in myocardial perfusion imaging using positron emission tomography. Eur. J. Nucl. Med..

[25] Bendriem B., Reed J., McCullough K., Khan M.R., Smith A.M., Thomas D., Long M. (2018). The continual innovation of commercial PET/CT solutions in nuclear cardiology: Siemens Healthineers. J. Nucl. Cardiol..

[26] Pan X.B., Declerck J., Burckhardt D. (2011). Cardiac Positron Emission Tomography: Overview of Myocardial Perfusion, Myocardial Blood Flow and Myocardial Flow Reserve Imaging.

[27] Armstrong I.S., Hayden C., Memmott M.J., Arumugam P. (2022). A preliminary evaluation of a high temporal resolution data-driven motion correction algorithm for rubidium-82 on a SiPM PET-CT system. J. Nucl. Cardiol..

[28] Hayden C. (2024). Cardio Direct: Time-of-Flight, Data-Driven Motion Correction (TOF DDMC) for PET Cardiac Imaging. https://marketing.webassets.siemens-healthineers.com/721ead9af0c323b6/e8c662ada997/siemens-healthineers_mi_pet-ct_biograph-trinion_whitepaper_cardio-direct.pdf.

[29] Slomka P.J., Alexanderson E., Jácome R., Jiménez M., Romero E., Meave A., Le Meunier L., Dalhbom M., Berman D.S., Germano G. (2012). Comparison of Clinical Tools for Measurements of Regional Stress and Rest Myocardial Blood Flow Assessed with ^13^N-Ammonia PET/CT. J. Nucl. Med..

[30] Yalcin H., Valenta I., Zhao M., Tahari A., Lu D.-Y., Higuchi T., Yalcin F., Kucukler N., Soleimanifard Y., Zhou Y. (2019). Comparison of two software systems for quantification of myocardial blood flow in patients with hypertrophic cardiomyopathy. J. Nucl. Cardiol..

[31] Oliveira J.B., Sen Y.M., Wechalekar K. (2019). Intersoftware variability impacts classification of cardiac PET exams. J. Nucl. Cardiol..

[32] Monroy-Gonzalez A.G., Juarez-Orozco L.E., Han C., Vedder I.R., García D.V., Borra R., Slomka P.J., Nesterov S.V., Knuuti J., Slart R.H.J.A. (2019). Software reproducibility of myocardial blood flow and flow reserve quantification in ischemic heart disease: A 13N-ammonia PET study. J. Nucl. Cardiol..

[33] Byrne C., Kjaer A., Olsen N.E., Forman J.L., Hasbak P. (2021). Test–retest repeatability and software reproducibility of myocardial flow measurements using rest/adenosine stress Rubidium-82 PET/CT with and without motion correction in healthy young volunteers. J. Nucl. Cardiol..

[34] Nesterov S.V., Sciagrà R., Orozco L.E.J., Prior J.O., Settimo L., Han C., Deshayes E., Dekemp R.A., Ryzhkova D.V., Gwet K.L. (2022). One-tissue compartment model for myocardial perfusion quantification with N-13 ammonia PET provides matching results: A cross-comparison between Carimas, FlowQuant, and PMOD. J. Nucl. Cardiol..

[35] Choueiry J., Mistry N.P., Beanlands R.S.B., Dekemp R.A. (2023). Automated dynamic motion correction improves repeatability and reproducibility of myocardial blood flow quantification with rubidium-82 PET imaging. J. Nucl. Cardiol..

[36] Haghayegh S., Kang H.-A., Khoshnevis S., Smolensky M.H., Diller K.R. (2020). A comprehensive guideline for Bland–Altman and intra class correlation calculations to properly compare two methods of measurement and interpret findings. Physiol. Meas..

[37] Koo T.K., Li M.Y. (2016). A Guideline of Selecting and Reporting Intraclass Correlation Coefficients for Reliability Research. J. Chiropr. Med..

