# Supplementary material for: Inter-Software Reproducibility of Quantitative Values of Myocardial Blood Flow and Coronary Flow Reserve Acquired by [13N]NH3 MPI PET/CT and the Effect of Motion Correction Tools
Source: Diagnostics (Basel). 2025 Mar 4;15(5):613. doi: 10.3390/diagnostics15050613 (PMC11898590; doi:10.3390/diagnostics15050613)
Supplement: Supplementary file 1 [file diagnostics-15-00613-s001.zip › diagnostics-3431428-supplementary.pdf]

FIGURES

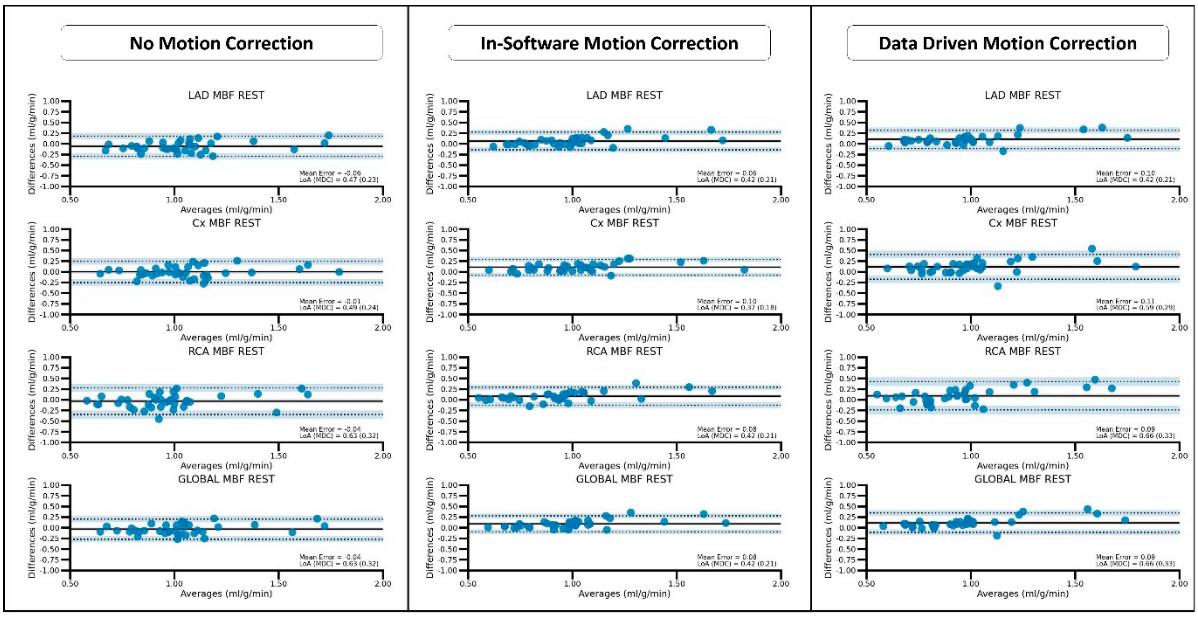

Figure S1. BA plots of the effect of motion correction (MC) tools in the agreement of MBF values during rest between QPET and 4DM.

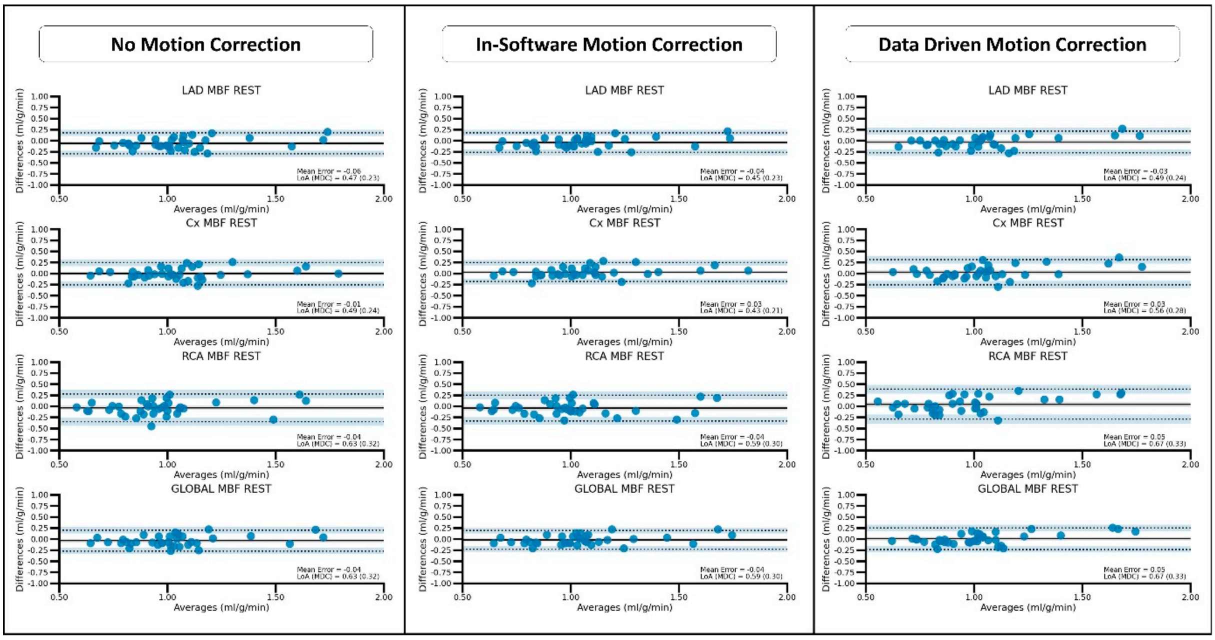

Figure S2. BA plots of the effect of motion correction (MC) tools in the agreement of MBF values during rest between QPET and Syngo.

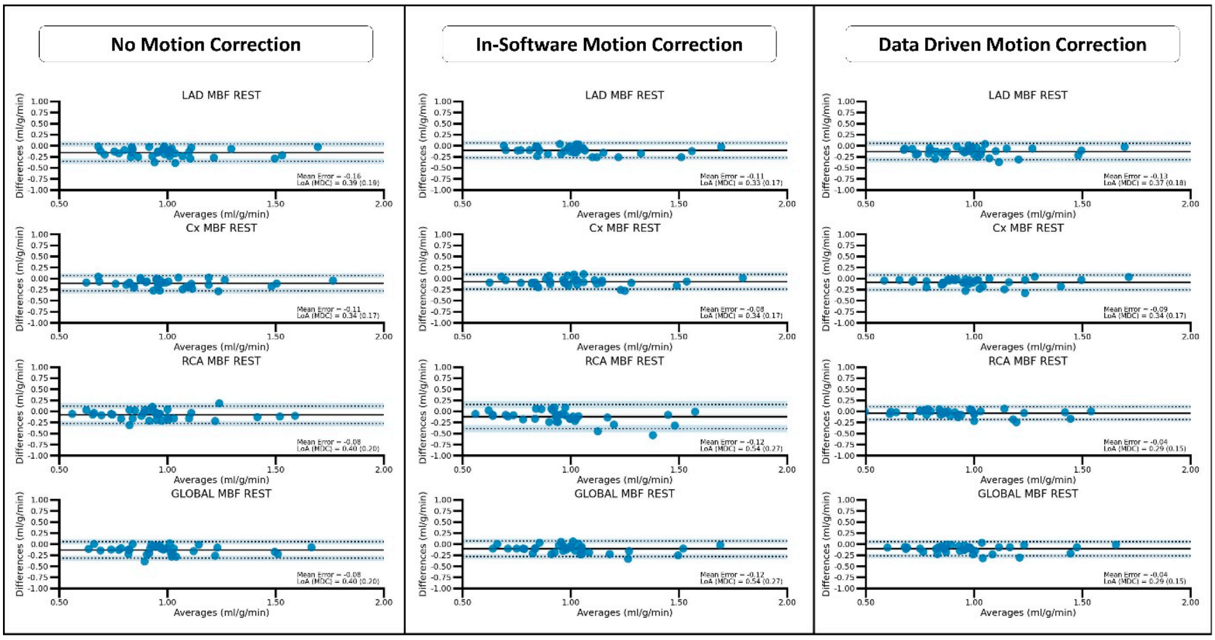

Figure S3. BA plots of the effect of motion correction (MC) tools in the agreement of MBF values during rest between 4DM and Syngo.

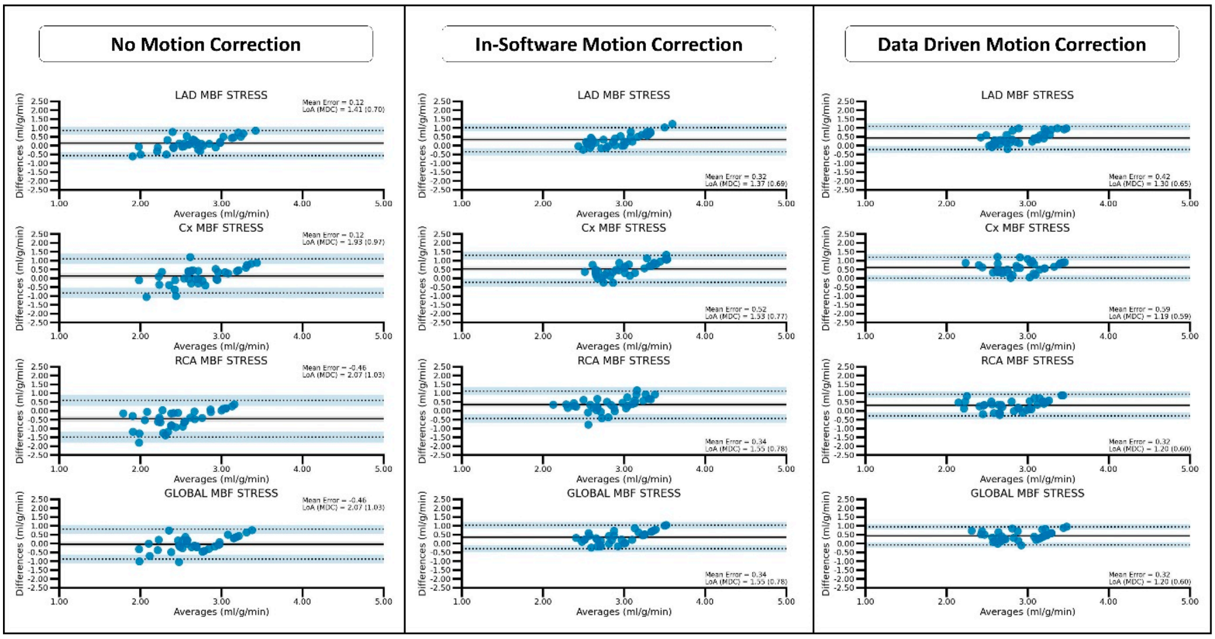

Figure S4. BA plots of the effect of motion correction (MC) tools in the agreement of MBF values during stress between QPET and 4DM.

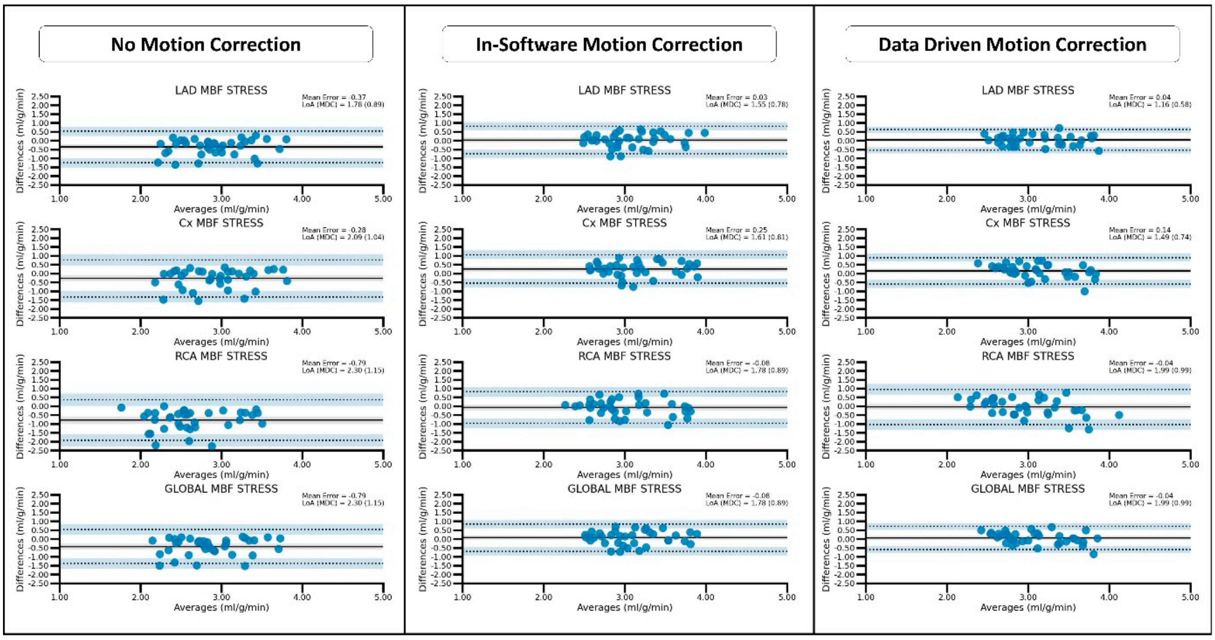

Figure S5. BA plots of the effect of motion correction (MC) tools in the agreement of MBF values during stress between QPET and Syngo.

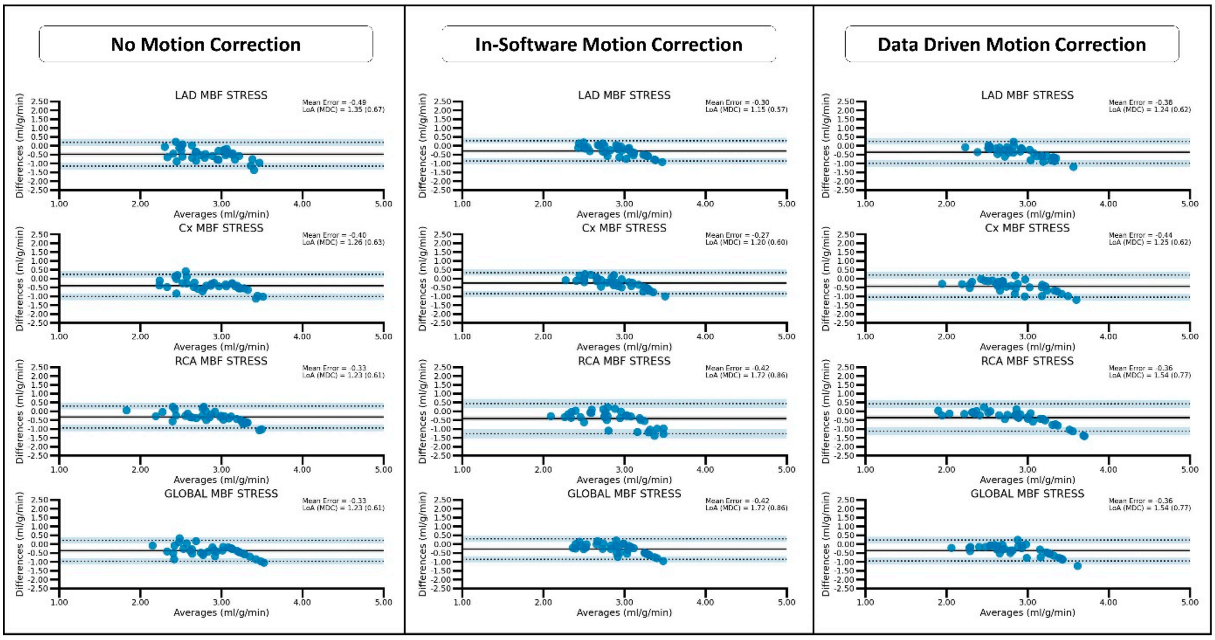

Figure S6. BA plots of the effect of motion correction (MC) tools in the agreement of MBF values during stress between 4DM and Syngo.

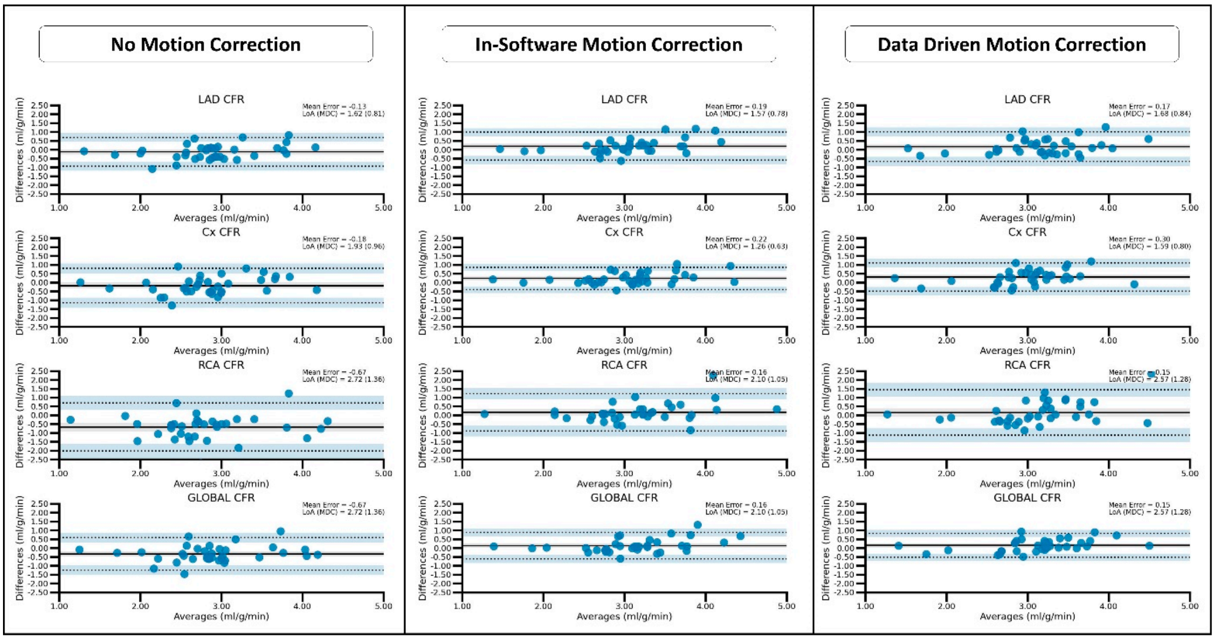

Figure S7. BA plots of the effect of motion correction (MC) tools in the agreement of CFR values between QPET and 4DM.

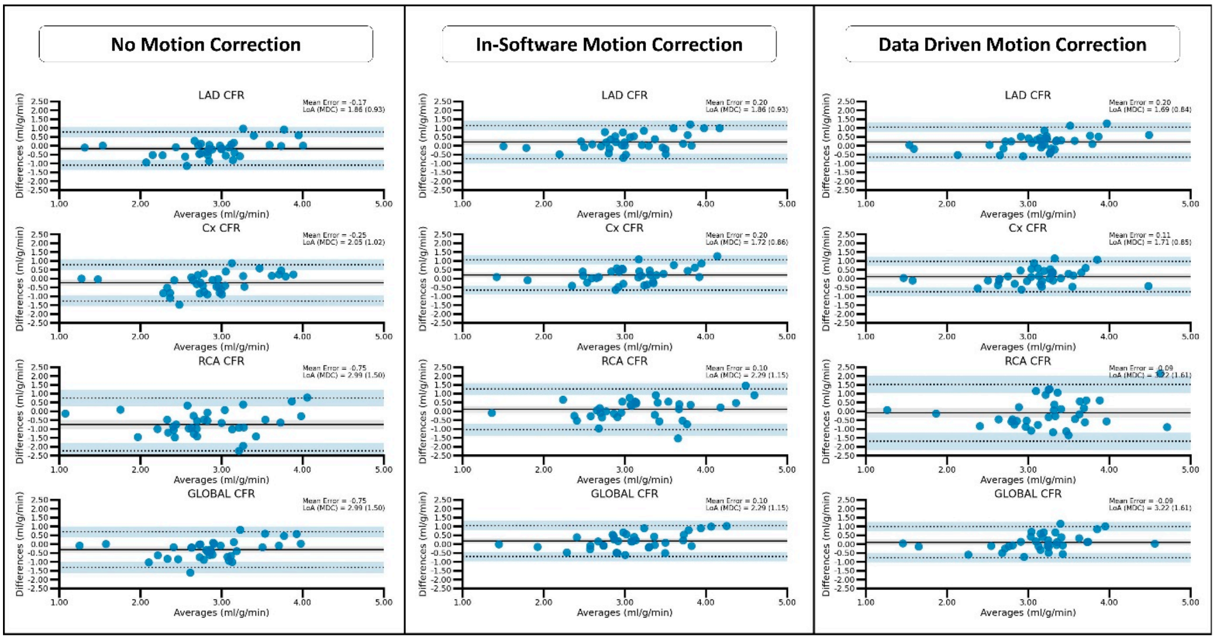

Figure S8. BA plots of the effect of motion correction (MC) tools in the agreement of CFR values between QPET and Syngo.

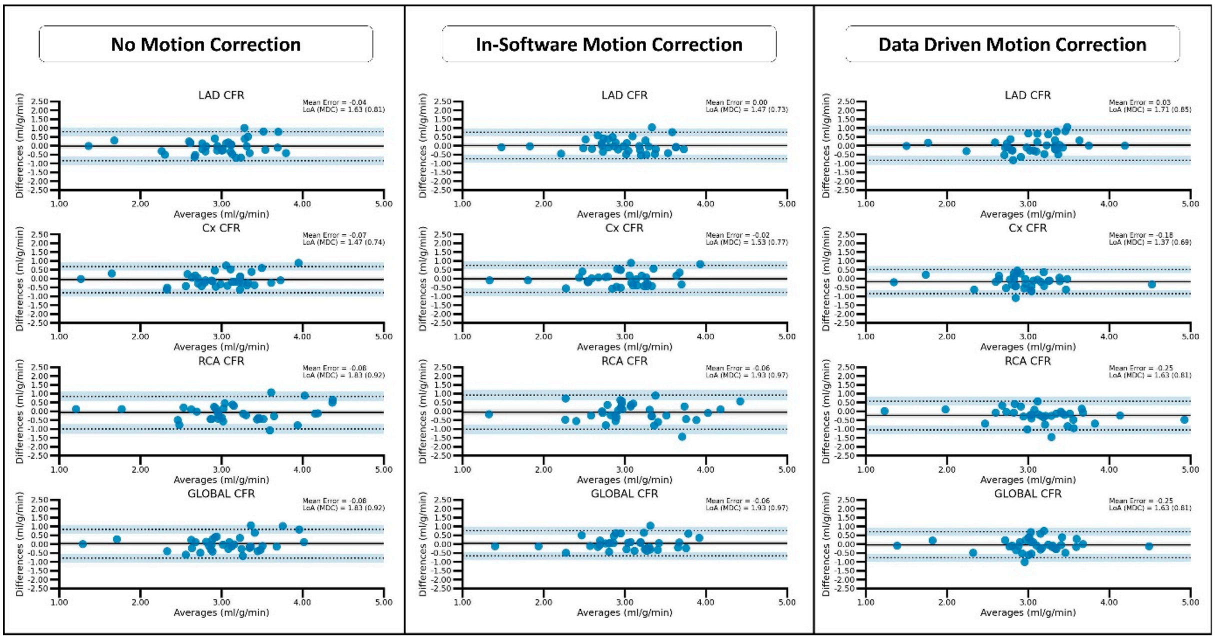

Figure S9. BA plots of the effect of motion correction (MC) tools in the agreement of CFR values between 4DM and Syngo.

RCA STRESS MBF

EFFECT SIZE OF MC TOOLS				INDIVIDUAL DIFFERENCES BETWEEN SPs		
EFFECT ISMC 4DM	EFFECT DDMC 4DM	EFFECT ISMC SYNGO	EFFECT DDMC SYNGO	INDIVIDUAL DIFFERENCES NMC	INDIVIDUAL DIFFERENCES ISMC	INDIVIDUAL DIFFERENCES DDMC
-0.19	-0.25	-0.06	-0.21	0.17	0.3	0.21
0	0.04	-0.25	0.43	0.02	0.23	0.41
-0.12	-0.24	0.11	0.07	0.26	0.03	0.05
0.01	-0.13	-0.01	0.19	0.27	0.25	0.59
-0.02	0	-0.46	0.14	0.3	0.14	0.44
-0.04	0.02	0.1	0.11	0.03	0.17	0.12
-0.38	-0.18	0.05	0.15	0.11	0.32	0.22
-0.42	-0.02	0.1	-0.55	0.64	1.16	0.11
-0.09	-0.3	-0.11	-0.67	0.35	0.33	0.02
-0.27	-0.33	-0.1	-0.31	0.26	0.09	0.24
0.09	-0.07	-0.14	-0.12	0.49	0.26	0.44
0.01	0.31	0.38	0.34	0.13	0.5	0.16
-0.07	-0.06	0.26	0.06	0.65	0.98	0.77
0.11	-0.08	0.19	-0.23	0.28	0.36	0.13
0.09	0.05	0.43	0.08	0.06	0.28	0.03
0.09	0.39	-0.14	0.38	0.33	0.1	0.32
-0.1	-0.13	-0.32	-0.33	0.25	0.03	0.05
0.43	0.04	-0.06	-0.39	0.57	0.08	0.14
-0.13	-0.06	1.26	0.15	0.01	1.38	0.2
-0.44	-0.8	-0.08	-0.83	0.26	0.62	0.23
-0.06	-0.31	0.01	-0.14	0.17	0.1	0
0.04	-0.24	-0.06	-0.44	0.4	0.3	0.2
-0.01	0.02	-0.16	0.09	0.65	0.5	0.72
-0.03	0.15	0.06	-0.01	0.24	0.33	0.08
-0.25	0.21	0.47	0.19	0.38	1.1	0.36
-0.08	-0.1	0	-0.19	0.31	0.39	0.22
0.16	0.2	-0.3	0.86	0.47	0.01	1.13
-0.13	0.03	-0.18	-0.54	1.08	1.03	0.51
0.01	0.01	-0.04	0.03	1.01	0.96	1.03
-0.01	0	-0.2	0.82	0.58	0.39	1.4
-0.15	0.01	0.64	0.89	0.49	1.28	1.37
-0.01	-0.07	0	-0.71	0.54	0.55	0.1
0.27	0.24	-0.02	0.24	0.34	0.05	0.34
-0.21	-0.12	0.27	-0.41	0.71	1.19	0.42
-0.05	0	-0.28	0.36	0.44	0.21	0.8
0.02	-0.08	0.03	-0.17	0.25	0.26	0.16

MEAN MBF NMC 4DM	2.70
MEAN MBF NMC SYNGO	3.02
MEAN INDIVIDUAL DIFFERENCE	0.38
DIFFERENCES IN MEAN VALUES	0.33
MEAN EFFECT ISMC 4DM	-0.05
MEAN EFFECT ISMC SYNGO	0.04
MEAN MBF ISMC 4DM	2.64
MEAN MBF ISMC SYNGO	3.06
MEAN INDIVIDUAL DIFFERENCE	0.45
DIFFERENCES IN MEAN VALUES	0.42
MEAN EFFECT DDMC 4DM	-0.05
MEAN EFFECT DDMC SYNGO	-0.02
MEAN MBF DDMC 4DM	2.65
MEAN MBF DDMC SYNGO	3.01
MEAN INDIVIDUAL DIFFERENCE	0.38
DIFFERENCES IN MEAN VALUES	0.36

4DM vs. Syngo
ICC
(95% CI)

NMC	ISMC	DDMC
0.72 (0.52 - 0.85)	0.49^{VV} (0.20 - 0.70)	0.71 (0.50 - 0.84)

Figure S10. Decrease in ICC after the use of MC. Note how when using ISMC the effect of the correction is opposite in both methods (the combination of green and red arrows). Observe how this change causes the mean individual difference (blue box) between the SPs to be higher after the use of ISMC than in the original NMC data. Compare to the DDMC approach, where the effect of the use MC was homogenous and the mean individual difference was relatively equal than when using NMC.

TABLES

Table S1. Regional and global spillover fractions of the study cohort

Patient ID	LAD	Cx	RCA	Global
1B	0.56	0.43	0.72	0.57
2B	0.55	0.57	0.78	0.61
3B	0.55	0.46	0.56	0.53
4B	0.58	0.64	0.78	0.65
5B	0.39	0.42	0.52	0.43
6B	0.53	0.44	0.58	0.52
7B	0.45	0.27	0.46	0.41
8B	0.52	0.52	0.59	0.53
9B	0.44	0.46	0.48	0.45
10B	0.66	0.59	0.64	0.64
1	0.51	0.53	0.65	0.55
2	0.57	0.52	0.74	0.60
3	0.39	0.40	0.56	0.43
7	0.44	0.41	0.70	0.49
8	0.47	0.43	0.57	0.49
10	0.41	0.29	0.41	0.38
12	0.49	0.39	0.60	0.49
13	0.48	0.44	0.54	0.48
18	0.46	0.34	0.48	0.43
19	0.44	0.37	0.58	0.45
20	0.37	0.32	0.56	0.40
21	0.54	0.44	0.42	0.49
22	0.47	0.44	0.52	0.47
24	0.35	0.27	0.50	0.36
26	0.42	0.33	0.38	0.39
27	0.39	0.35	0.48	0.40
31	0.37	0.29	0.53	0.39
33	0.35	0.20	0.49	0.35
34	0.52	0.43	0.56	0.51
35	0.42	0.44	0.71	0.50
36	0.46	0.35	0.56	0.45
37	0.53	0.58	0.77	0.60
38	0.57	0.61	0.67	0.61
40	0.55	0.51	0.51	0.53
42	0.61	0.62	0.74	0.64
44	0.42	0.39	0.45	0.43
